# PyUAT: An open-source Python framework for uncertainty-aware, efficient, and scalable model-driven cell tracking

**DOI:** 10.1371/journal.pone.0337110

**Published:** 2025-12-11

**Authors:** Johannes Seiffarth, Katharina Nöh

**Affiliations:** 1 Institute of Bio- and Geosciences, IBG-1: Biotechnology, Forschungszentrum Jülich, Jülich, Germany; 2 Computational Systems Biotechnology (AVT.CSB), RWTH Aachen University, Aachen, Germany; Universitat Pompeu Fabra, SPAIN

## Abstract

Tracking individual cells in live-cell imaging provides fundamental insights into phenotypic heterogeneity and cellular responses to environmental change. However, microbial cell tracking is particularly challenging, as cell growth is characterized by stochastic cell movements and frequent divisions, while time-lapses are recorded at limited frame rates to avoid counterfactual results. Here, we investigate how probabilistic Uncertainty-Aware Tracking (UAT), a paradigm based on statistical models of cell behavior, robustifies tracking quality under such challenging conditions. Using , the first open-source implementation of UAT, we systematically analyze the role of cell development models on tracking quality under increasing imaging intervals. Our results on a large 2D+t dataset demonstrate that model-driven cell tracking not only achieves higher accuracy at low frame rates, but also outperforms comparable methods in runtime efficiency. is available at https://github.com/JuBiotech/PyUAT, including example notebooks for immediate use in Google Colab.

## Introduction

Microfluidic live-cell imaging (MLCI) is an emerging high-throughput technology for monitoring the spatio-temporal development of microbial cells under precisely controllable conditions, with hundreds of replicates per experiment [1,2]. Due to its ability to record the development of individual cells within 2D monolayer cavities, MLCI is ideally suited to study the causes and consequences of phenotypic heterogeneity that occurs within isogenic microbial populations and consortia. This capability has been proven to be highly informative, as evidenced by diverse applications in biomedical, biotechnological, and ecological fields [3–5]. For example, MLCI has provided unique quantitative insights into the phenotypic heterogeneity of microbial organisms in constant and fluctuating environments [6–8], responses to exposure to stress factors [9], or the impact of biological noise [10].

**Fig 1:**
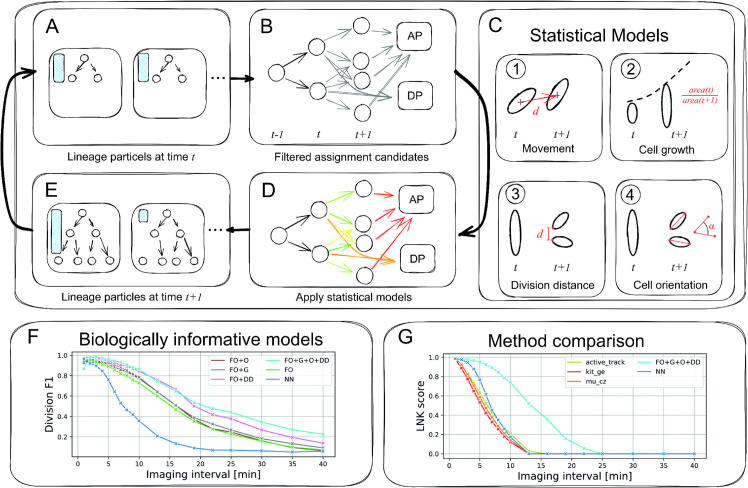
Schematic overview of the UAT workflow steps (A to E), and tracking performance comparison (F, G). In (A+E), the size of the blue boxes indicates the probability of a lineage particle (the larger, the more likely). The edge colors in B and D indicate that non-scored (grey) and probability-scored assignments (green – high, yellow – medium, red – low probability). AP and DP denote appearance and disappearance assignments, respectively. (F) shows derived division *F1* scores for different statistical model compositions and increasing imaging intervals. (G) compares the tracking quality (see text) of with established tracking methods, measuring the *LNK* score at various imaging intervals (median of five time-lapse sequences).

To gain insight into the development of colonies, accurate cell pedigrees spanning several generations need to be extracted from the time-lapse images. This information is captured in cell lineage trees (CLT), which are bifurcated trees with the cell instances serving as nodes and the edges representing the frame-to-frame associations of the cells, with a branch indicating a cell division (Fig 1). The components of generating CLTs are, thus, the segmentation of individual cells in each image and the tracking of these cells throughout the time-lapse. Today, high-quality deep-learning (DL) segmentation models are available for the microbial domain, providing accurate segmentation results across organisms and imaging modalities [11–13]. In contrast, microbial cell tracking is generally considered more complicated because cells exhibit high visual similarity at low temporal resolution [14]. DL-based tracking solutions have only recently been proposed for 1D micro-channels (also known as mother machines) and 2D micro-chambers [15,16]. Nevertheless, ground truth tracking data is rare in the microbial domain. The lack of sufficient annotated data explains why classical (non-DL) linear assignment problem (LAP)-based trackers are still predominant. These trackers predict edges in a two-step procedure: first, the costs for potential edge candidates are determined from cell features (e.g., distance or mask overlap), and then edges with minimal costs are selected to link cells between frames. This process is repeated for each pair of subsequent frames to obtain the complete CLT.

In the microbial domain, the growth behavior within colonies is often stochastic, which implies that high frame rates are required to resolve cell tracks unambiguously. Although existing LAP cell trackers have been successfully used in other contexts, in practice their tracking quality deteriorates when imaging frequencies that are not tuned to the colony development rate are applied. For situations where the frame rate needs to be limited, for example, to avoid exposing cells to phototoxic stress and perform imaging with a "bio-safe" frame rate, [17] introduced the Uncertainty Aware Tracking () paradigm, a Bayesian multi-hypothesis cell tracking framework that incorporates knowledge of temporal cell features, such as cell elongation rates or division angles, into explainable statistical models that improve the quality of CLT inference. Notably, the statistical models are able to learn from past cell behavior and use this knowledge to make informed track predictions, giving the models a self-learning capacity. Unlike LAP cell trackers, generates a distribution of CLTs that represents the uncertainty inherent in the CLT generation process, allowing to account for potentially many CLTs in the interpretation of the tracking results [17]. However, the Bayesian UAT ensemble approach comes at the cost of a much higher computational effort.

Although the Bayesian approach provides a principled framework for CLT reconstruction, the high computational cost limits its practical applicability to large datasets with many cell detections and division events. To retain the advantages of UAT while making it compatible with microbial live-cell imaging applications, two key challenges must be addressed:

1. optimizing the runtime efficiency of the CLT reconstruction process to ensure its scalability, and
2. enabling the implementation of tailored cell development models and model combinations.

To meet these requirements, we developed the Python package , designed for efficiency and modular integration of custom cell development models. We demonstrate the scalability of by performing cell tracking in MLCI time-lapses containing 100k+ cell instances. We then compare the tracking performance of with that of recent comparable LAP trackers. We examine the efficacy of the statistical models and model compositions, which are at the core of the UAT paradigm. We systematically investigate the tracking performance at lower imaging rates, and thereby reveal the importance of modeling specific cell behaviors for cell tracking.

## Approach and implementation

performs iterative frame-to-frame cell tracking based on Bayesian multi-hypotheses tracking (MHT) using a particle filter (Fig 1A–E). First, a distribution of CLT hypotheses (particles) is given at frame *t* (A), where initially these particles represent empty CLTs. For each of these hypotheses, all possible assignment candidates are generated that link cells between the current (*t*) and the next frame (*t*  +  1) (B). Every assignment candidate is awarded a likelihood using biologically informed statistical models (C-D). Solving an integer linear program (ILP) yields the set of most likely assignments and extends the existing particles. Based on the set of most likely frame-to-frame extensions, new particles are sampled to form the updated CLT distribution at frame *t*  +  1, instantiating a self-learning capacity (E). This procedure is repeated for every pair of consecutive frames in the time-lapse, resembling the particle filter that finally yields the posterior probability distribution of CLTs. For a mathematical description of the Bayesian MHT approach and the particle filter, we refer to [17].

We here focus on two core elements of the algorithm, the formulation of biologically informed statistical assignment models and the efficient solution of the assignment problem. In , four types of assignment link cells between consecutive frames: cell appearance, disappearance, migration, and cell division. The cell appearance and disappearance assignments describe the creation of new cells and the end of cell tracks, respectively. These assignments are used to deal with cells that appear or disappear from the field of view of the image, as well as segmentation artifacts. Migration and cell division assignments model cell movement and division into daughter cells. For each type of assignment, we define a set of statistical models (denoted *assignment models*) that score assignments according to the likelihood of known single-cell features. For example, the cell area growth model gives the likelihood of the increase in cell size within a given period of time. Further statistical models capture knowledge about cell movement, division distance and orientation. In addition, a custom model can be designed in the modular framework. The four assignment models (one for each assignment type) build a tracking configuration.

Particular single-cell features are modeled using univariate probability density distributions, such as (half-)normal distributions or kernel density estimates (S1 Appendix), which are specified in SciPy with parameters chosen based on biological knowledge [18]. For example, we model the growth rate of the single cell area - the rate each cell increases its size - using a half-normal distribution with the growth rate of the colony as the mean, and empirically select a variance that accounts for the expected variation of the single cell. A detailed guide for selecting model parameters is outlined in (S2 Appendix). The univariate PDF models are combined to a joint distribution that forms the assignment model.

The particle filter then assesses the assignment candidates at frame *t* according to the likelihood provided by the underlying assignment models to sample the CLT distribution for frame *t* + 1. Importantly and unlike existing LAP trackers, our UAT approach takes advantage of all single-cell features based on the CLT up to time *t*. This allows building powerful self-learning statistical models in a modular, Lego-like fashion that take advantage of the past cell development information, and which is utilized to predict future lineage development.

Computationally, scoring assignments based on the statistical assignment models relies on the computation of single-cell features, extracted from the CLT up to frame *t*, such as the movement of a cell in past frames. Computing these features for every cell requires traversing all CLT hypotheses and needs to be repeated in every frame-to-frame iteration. To efficiently traverse the CLT hypotheses for thousands of cells and aggregate their information along their temporal development, we developed NumPy array based walks through the CLTs utilizing NumPy’s efficient and vectorized computations (S3 Appendix). These NumPy arrays are efficiently distributed among parallel processes using Ray [19]. To further improve efficiency, we filter for sensible assignment proposals, such as limiting the displacement radius of cells between subsequent frames. All statistical models are evaluated utilizing the vectorized implementation of SciPy distributions.

Based on the set of scored assignments, constructs an ILP to sample likely frame-to-frame extensions. The objective function of the ILP consists of the assignment scores and is optimized subject to linear constraints ensuring the validity of the lineage solutions (S4 Appendix). For solving ILPs, proprietary (Gurobi, default) or open-source (Cbc, https://github.com/coin-or/Cbc) optimizers are available in . Gurobi is the default due to faster optimization performance, while Cbc is an open-source solution that works out of the box. Computations are accelerated by multi-process optimization of the ILP solver or, optimally, by parallel computation of multiple CLT particles.

## Results and discussion

We here evaluate in two steps: First, we use its modular implementation to design biologically motivated models that capture typical cell behavior and investigate their importance for high-quality tracking under increasing imaging intervals. Second, we compare ’s tracking quality and execution times with three recent non-DL LAP tracking methods. For the evaluation, we use a public dataset consisting of five manually curated time-lapse sequences of recorded for more than 13 h, with one image taken every minute [20]. In total, the dataset contains 1.4 million cell detections that are linked into more than 29k cell tracks. To challenge the tracking methods, larger imaging intervals are generated by sub-sampling in time (see dataset sizes in S5 Appendix).

### Evaluation of tailored statistical tracking models at varying frame rates

Taking advantage of the modularity of , we build univariate models that capture specific single-cell features and assemble these models into assignment and tracking configurations to investigate their effectiveness in tracking cells at decreasing frame rates. First, we design a baseline nearest neighbor configuration (NN) assuming zero cell motion and growth between consecutive images. For the second configuration, we assume that cells preserve their movement and cell area growth rate and derive their movement and growth from cell development in the past to predict future cell positions and areas. We term this the “first order” model (FO). In both cases, we model the difference between predicted and observed cell features using a half-normal and normal distribution. Moreover, we introduce cell growth (G), cell orientation (O) and division distance assignment (DD) models that incorporate biological knowledge specific to the studied organism. For the G model, we estimate the mean single-cell area growth rate based on the colony growth (segmentation only), and model its variability using a normal distribution. The O model captures the “snapping” division behavior of and models the angle between the major axes of the two daughter cells using a normal distribution. Similarly, the rotation angles between cells in a migration assignment are modeled. Finally, the division distance of two daughter cells is modeled using a half-normal distribution. Details about the statistical distributions are given in S1 Appendix, where Table S1.1 summarizes the six tracking configurations that we study in the following, i.e., NN, FO, FO+O, FO+G, FO+DD, and FO+O+G+DD.

Fig 1F shows the tracking performance of the six configurations for a range of imaging intervals, measured using the *F1* division scores, which describe the amount of correctly reconstructed cell divisions. The *F1* score is computed using traccuracy (https://github.com/Janelia-Trackathon-2023/traccuracy). The NN baseline shows high division reconstruction at low imaging intervals, but the quality decreases rapidly with lower frame rates. The FO configuration yields much better division *F1* scores, while being slightly improved by adding cell orientation (FO+O) and growth (FO+G) models. Using the division distance assignment model (FO+DD) enforces daughter cells of divisions to have an empirically observed close spatial distance and increases the tracking quality across a wide range of imaging intervals. Combining all models into a single composite tracking configuration (FO+G+O+DD) shows similar division reconstruction performance but outperforms FO+DD at longer imaging intervals, thus effectively utilizing the joint information of the univariate statistical models. The two models with the biggest improvement in division reconstruction are FO and DD. Thus, the ability to learn information about past cell behavior and explicitly model cell division is crucial for high-quality CLT inference.

### Comparison to existing tracking methods

To investigate the tracking performance of our implementation, we select three recent non-DL tracking methods, namely MU_CZ, KIT_GE (Cell Tracking Challenge nomenclature) and ActiveTrack [21,22]. MU_CZ measures the overlap between the segmentation masks of consecutive frames to greedily link cells (https://celltrackingchallenge.net/participants/MU-CZ/, Version: 2). KIT_GE utilizes the graph structure of tracking and represents the tracking task as a coupled minimum cost flow problem based on cell detections [21]. ActiveTrack measures the “activity” of cells in consecutive images to predict cell migration [22]. These tracking methods are executed using their default parameter values. The time-dependent tracking parameters of are adjusted to the imaging interval. The tracking quality of the computed CLTs is measured using the Cell Tracking Challenge (CTC, https://celltrackingchallenge.net/) *LNK* score, which describes the overall quality of the tracking (0 worst, 1 best), computed using traccuracy (https://github.com/Janelia-Trackathon-2023/traccuracy).

Fig 1G shows the *LNK* tracking score with the given segmentation ground truth. Cell tracking algorithms are compared with the baseline NN and the best-performing FO+G+O+DD tracking configuration. In Fig 1G, we observe a strong decrease in the *LNK* score for all methods at longer imaging intervals. Whereas the NN configuration performs similarly to three selected tracking methods and collapses at 16 min intervals, the FO+G+O+DD configuration consistently outperforms all other tracking methods, especially at longer imaging intervals, eventually collapsing at 25 min. Thus, the tailored statistical models and their combined biological knowledge enable to perform more robust tracking up to moderate imaging intervals.

### Comparison of tracking execution times

Finally, we compare the execution times of for the six configurations and compare the fastest and slowest configuration with those of MU_CZ, KIT_GE, and ActiveTrack. The execution times are measured on a system equipped with 2x AMD EPYC 7282 16-core processor and 504 GB. All tracking methods are executed on a single core, while the evaluation of the different tracking methods and imaging intervals is run in parallel batches of 32. The execution time includes data loading, storing, and the tracking runtime.

**Fig 2:**
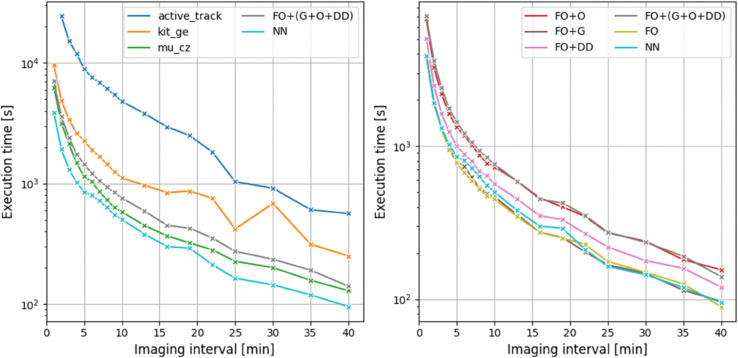
Execution times for varying imaging intervals of and the MU_CZ, KIT_GE and ActiveTrack tracking methods (left), as well as among the different tracking configurations (right).

The results are shown in Fig 2. Clearly, processing lower frame rates generally leads to a reduction in execution time, as the tracking is performed for fewer images and, therefore, fewer cell detections. Notably, our efficient implementation and assignment scoring makes the fastest of all tracking methods considered when using the NN configuration, while the composite FO+G+O+DD configuration is only slightly outperformed in execution time by the greedy MU_CZ method. Although the composite model is substantially more accurate at long imaging intervals (Fig 2G), as measured by the *LNK* score, its execution times are comparable to those of existing trackers. Specifically, performs the tracking in at most 2 hours, which is only a fraction of the recoding time of 13.3 h for each time-lapse.

## Conclusion

The Python package is the first open-source implementation of the paradigm in an efficient and modular framework. Its design enables the development of customized, interpretable statistical models with self-learning capabilities to predict future cell behavior, distinguishing the approach from existing tracking methods. Using ’s modular model composition, we have investigated the role of individual cell development models and demonstrated their impact on tracking quality and robustness. Under challenging conditions, such as limited frame rates, not only delivers more accurate tracking results, but also achieves faster runtimes than comparable non-DL tracking methods. The flexibility of the framework allows for adaptation and the design of new assignment models beyond the studied cell organism. Integration of available biological knowledge makes more robust compared to existing methods, especially when labeled training data are scarce. Furthermore, its efficient implementation enables lineage tree reconstruction in a fraction of the experiment time. Together, these features make a versatile and explainable cell tracking tool and we expect it to provide a foundation for future cell tracking benchmarks in microbial live-cell imaging.

## Supporting information

S1 Appendix Scoring assignments using statistical models. (PDF)

S2 Appendix Choosing statistical model parameters. (PDF)

S3 Appendix Tensor walks. (PDF)

S4 Appendix Computing optimal frame-to-frame lineages. (PDF)

S5 Appendix Sub-sampled dataset size statistics. (PDF)
