# Supplementary material for: PyUAT: An open-source Python framework for uncertainty-aware, efficient, and scalable model-driven cell tracking
Source: PLoS One. 2025 Dec 11;20(12):e0337110. doi: 10.1371/journal.pone.0337110 (PMC12697953; doi:10.1371/journal.pone.0337110)
Supplement: S1 Appendix — (PDF) [file pone.0337110.s001.pdf]

Supplementary Information S1 for

PyUAT: An open-source Python framework for uncertainty-aware, efficient, and scalable model-driven cell tracking

Johannes Seiffarth [1,2] and Katharina Nöh [1,*]

[1] Institute of Bio- and Geosciences, IBG-1: Biotechnology, Forschungszentrum Jülich, 52425 Jülich, Germany

[3] Computational Systems Biotechnology (AVT.CSB), RWTH Aachen University, 52062 Aachen, Germany

[*]Correspondence: k.noeh@fz-juelich.de

S1.1 Scoring assignments using statistical models

`PyUAT` uses statistical models to score assignments that connect cell detections in consecutive frames using appearance, disappearance, migration and cell division assignments. Given a possible assignment of a specific type, a set of models is used to compute the assignments' likelihood. `PyUAT` allows to customize the set of statistical models and tailor them to the specific behavior of the biological organism. The computed assignment likelihoods are then used in the particle filter for sampling from the lineage distribution or determining the most likely lineage.

For `PyUAT` we developed various models that score appearance or disappearance of cells, their spatial movement, cell growth and orientation. In the following, we give the mathematical definitions of these models and show how to use them for computing probabilities for assignments.

Notation

Let $\mathbb{D} = \{D^1, \cdots, D^T\}$ be the set of all cell detections in a time-lapse of length $T \in \mathbb{N}$ where $D^t = \{d_1^t, \cdots d_{N_t}^t\}$ is the set of $N_t \in \mathbb{N}$ cell detections at frame t. An assignment is defined as a tuple that consists of cell detections of two consecutive frames. For the four assignment types, we denote the set of all possible assignments between frames t and $t+$ as $A_{t:t+1}$. For an assignment $a \in A_{t:t+1}$, we introduce the following notation:

- An appearance assignment models the appearance of a cell detection at frame $t+1$ that has no predecessor in frame t. We denote the assignment as
$$a := \left(\emptyset, \{d^{t+1}\}\right), \quad d^{t+1} \in D^{t+1}$$
- Similarly, the disappearance assignment contains a cell detection at frame t that has no successor in frame $t+1$:
$$a := \left(\{d^t\}, \emptyset\right), \quad d^t \in D^t$$
- The migration assignment connects a cell detection at fame t to a cell detection at the next frame $t+1$
$$a := \left(\{d^t\}, \{d^{t+1}\}\right), \quad d^t \in D^t,\ d^{t+1} \in D^{t+1}$$
- The cell division assignment captures the division of a single cell into two daugther cells and connects a single cell detection at frame t to two cell detections at the next frame $t+1$
$$a := \left(\{d^t\}, \{d_1^{t+1}, d_2^{t+1}\}\right), \quad d^t \in D^t,\ d_1^{t+1}, d_2^{t+1} \in D^{t+1}$$

For brevity, we omit the set-brackets from the assignment notation. For instance, a cell division assignment is expressed as

$$a := \left(d^t; d_1^{t+1}, d_2^{t+1}\right) \tag{S1.1}$$

Furthermore, ϕ_{norm} and ϕ_{hn} denote the probability density function (PDF) of a normal and half-normal distribution, respectively.

Constant probability model (CP)

The simplest model for scoring assignments is a constant probability model. Without extracting a single-cell quantity, this model will always return a constant probability.

$$p(a) = c, \quad c \in [0, 1] \tag{S1.2}$$

where a is an assignment and c is the constant probability. We use the model mainly to score appearance and disappearance assignments. These events happen when cells leave the microscopy's field of view or due to errors in the segmentation.

Nearest neighbor model (`NN`)

For slow cell growth behavior, where cells do not move much nor change their size between consecutive images, an assignment model should give high probability to assignments where cells stay at the same position and maintain their size (nearest neighbor). For migration assignments, we directly compare the position and cell size, whereas for division assignments, we compute the center of mass and joint size to compare it with the position and cell size of the previous frame. We utilize a half-normal distribution with mode 0 (no movement) to model the distance between linked cells and a positive scale parameter defining the variance of the distribution. The relative size change for the growth model is scored using a normal distribution with a mean of 1 (no growth) and a positive scale parameter.

Thus, for the movement and growth models we compute the likelihood for an assignment $a = (d^t, d^{t+1}) \in A_t$:

$$p(d^t, d^{t+1}) = \phi_{hn}\left(dist\left(d^t, d^{t+1}\right)\right) \cdot \phi_{norm}\left(\frac{area(d^{t+1})}{area(d^t)}\right) \tag{S1.3}$$

where $dist(\cdot, \cdot)$ denotes the Euclidean distance of the two linked detections and $area(\cdot)$ the size of the detection, respectively. For positions, this is the Euclidean distance between the centers of the two cell detections, and for cell sizes, this is the relative area change (growth).

Similarly, for cell division models, we define

$$p(d^t; d_1^{t+1}, d_2^{t+1}) = \phi_{hn}\left(dist\left(d^t; d_1^{t+1}, d_2^{t+1}\right)\right) \cdot \phi_{norm}\left(\frac{area(d_1^{t+1}) + area(d_2^{t+1})}{area(d^t)}\right) \tag{S1.4}$$

where $dist(\cdot; \cdot, \cdot)$ denotes the Euclidean distance of the parent cell to the center of mass of the progeny.

First-order models (`FO`): Predict movement & Predict growth

`PyUAT` has access to the complete CLT and the temporal evolution of cell properties during its iterative tracking procedure. The prediction models utilize this knowledge about the temporal development of single-cell quantities up to frame t for predicting their development for the frame $t + 1$.

The residual between a predicted single-cell quantity and a actually observed cell quantity at time $t + 1$ is computed and scored using a statistical distribution. Both quantities (position and area) are scored using a half-norm distribution with mode 0 (exactly predicted cell position/size) and a positive scale parameter.

Thus, similar to the `NN` model, we write the migration probability

$$p(d^t, d^{t+1}) = \phi_{hn}\left(dist(pred(d^t), d^{t+1})\right) \tag{S1.5}$$

and cell division probability

$$p(d^t; d_1^{t+1}, d_2^{t+1}) = \phi_{norm}\left(dist\left(pred(d^t); d_1^{t+1}, d_2^{t+1}\right)\right) \tag{S1.6}$$

where $pred(\cdot)$ predicts the cell property at frame $t+1$, and $dist(\cdot,\cdot)$ or $dist(\cdot;\cdot,\cdot)$ computes the element-wise distance in the cell's feature space. The prediction of cell property is performed by averaging the cell property over a time span of $n \in \mathbb{N}$ frames. Therefore, we perform walks of length $n \in \mathbb{N}$ along the cell lineage tree (CLT) using the `tensortree` library (see S3_Appendix). Longer walks may provide more robust predictions while short walks prioritize the most recent development. In this work, we used short walks of $n = 1$. Notice, that for a walk length of $n = 0$, the models are equivalent to the no movement/growth models in Sec. Nearest neighbor model.

Orientation models (O)

Living *C. glutamicum* cells usually change their orientation only slowly throughout their development. We quantify this change in orientation by the angle between the major-axis of the rod-shaped cells in consecutive images. For cell migration the angle changes slowly due to cell growth and displacement and is modeled using a half-normal distribution with a mean of 0 and a positive scale parameter. However, during cell division, *C. glutamicum* exhibits a "snapping" behavior such that the daughter cells end up in a characteristic angle towards each other. Therefore, for cell division events, we model the angle of the daughter cells using a normal distribution with a mean of 135 degrees and a positive scale parameter.

Thus, the likelihood of a migration assignment is defined as

$$p(d^t, d^{t+1}) = \phi_{norm}\left(angle\left(d^t, d_{t+1}\right)\right) \tag{S1.7}$$

and for a cell division assignment by

$$p(d^t; d_1^{t+1}, d_2^{t+1}) = \phi_{norm}\left(angle\left(d_1^{t+1}, d_2^{t+1}\right)\right) \tag{S1.8}$$

where angle$(\cdot,\cdot)$ denotes the angle between the major axes of two cell detections.

Division distance model (DD)

When cells divide into two daughter cells, the progeny are usually very close together, with no cell between them. We utilize this observation and model the distance between daughter cells using a half-norm distribution with mode 0 and a positive scale parameter.

$$p(d^t; d_1^{t+1}, d_2^{t+1}) = \phi_{norm}\left(dist\left(d_1^{t+1}, d_2^{t+1}\right)\right) \tag{S1.9}$$

where $dist(\cdot,\cdot)$ denotes the minimum Euclidean distance between the major axes of two cell detections.

Biological growth model (G)

For microbial organisms, usually at least some information about their growth capabilities is available for the studied cultivation conditions. However, if this is not the case, we can still utilize the segmentation information to quantify the growth rates of the microbial colony and use this information as an estimate for the single-cell growth rates. In this work, we choose mean values of 1.008 and 1.016 as the expected growth rate for migration and cell division assignments, respectively. Using a normal distribution with these mean values, the probability of the assignments using the growth model is computed as follows for migration

$$p(d^t, d^{t+1}) = \phi_{norm}\left(\frac{area(d^{t+1})}{area(d^t)}\right) \tag{S1.10}$$

and division assignments

$$p(d^t; d_1^{t+1}, d_2^{t+1}) = \phi_{norm}\left(\frac{area(d_1^{t+1}) + area(d_2^{t+1})}{area(d^t)}\right) \tag{S1.11}$$

When subsampling the images, the temporal distance between the images is increased and larger frame-to-frame cell size growth is to be expected between consecutive frames. In our exponential growth model, we compensate by introducing an exponential subsampling factor. For a subsampling factor $\tau \in \mathbb{N}$, we choose mean $\bar{\mu}_{\text{migration}}$ and $\bar{\mu}_{\text{division}}$ for migration and cell division assignments:

$$\bar{\mu}_{\text{migration}} = 1.008^{\tau}, \quad \bar{\mu}_{\text{division}} = 1.016^{\tau} \tag{S1.12}$$

S1.2 Computing probabilities from densities

The half-normal or normal distributions used for assignment models do not provide probabilities, but probability densities. For computing probabilities, we need to integrate the probability density function (PDF). Therefore, we define the probability of a measured quantity (e.g. size change, position difference) as the probability of more extreme quantities. Let $q \in \mathbb{R}$ be the quantity extracted for a specific assignment a, and let $\phi(\cdot)$ be the PDF for an assignment model. Then the probability of assignment a is defined as:

$$p(q) = 1 - \int_{M-\delta}^{M+\delta} \phi(x;\theta)\ dx \tag{S1.13}$$

where M is the mode of the distribution, θ are distribution parameters that define the distribution's PDF, and $\delta = |M - q|$ is the distance between mode and the measured quantity.

Half-normal densities

For half-normal PDFs $\phi_{hn}(\cdot)$ with mode $M = 0$, scale parameter $\sigma \in \mathbb{R}_{>0}$, and observed quantity $q \in \mathbb{R}_{\geq 0}$, the probability is computed as:

$$\begin{aligned} \phi_{hn}(q) &= 1 - \int_{M-\delta}^{M+\delta} \phi_{hn}(x;\sigma)\ dx = 1 - \int_{0-|0-q|}^{0+|0+q|} \phi_{hn}(x;\sigma)\ dx = 1 - \int_{-q}^{+q} \phi_{hn}(x;\sigma)\ dx \\ &= 1 - \int_{0}^{q} \phi_{hn}(x;\sigma)\ dx. \end{aligned} \tag{S1.14}$$

Normal densities

For normal PDFs $\phi_{norm}(\cdot)$, mean $M = \mu$, scale parameter $\sigma \in \mathbb{R}_{>0}$, and observed quantity $q \in \mathbb{R}$, the probability is computed as:

$$\begin{aligned} p_{norm}(q) &= 1 - \int_{M-\delta}^{M+\delta} \phi_{norm}(x;\sigma)\ dx = 1 - \int_{\mu-\delta}^{\mu+\delta} \phi_{norm}(x;\sigma)\ dx \\ &= \int_{-\infty}^{\mu-\delta} \phi_{norm}(x;\sigma)\ dx + \int_{\mu+\delta}^{\infty} \phi_{norm}(x;\sigma)\ dx. \end{aligned} \tag{S1.15}$$

S1.3 Tracking configurations

The assignment models described above compute likelihoods for a specific property of the assignment, for instance, the cell movement or growth rate. To score multiple properties, we define sets of these assignment models for every type of assignments, that is, appearance and disappearance assignments, migration and cell division assignments. Table S1.1 gives an overview of the designed tracking configurations and the models involved for computing assignment likelihoods. The `NN` configuration utilizes the constant probability model to score appearance and disappearance assignments (used in all other configurations, too). The migration and cell division assignments are scored with the "no movement" and "no growth" models. The first-order (`FO`) configuration utilizes the first-order models to predict and score movement and cell size changes. We utilize the `FO` model as a baseline and combine all the following configurations with this configuration. The `FO`+orientation model adds the orientation models to the configuration. The `FO`+distance model adds the cell division distance model to the configuration, and `FO`+growth model overwrites the `FO` growth model and utilizes the growth model based on biological prior knowledge.

Table S1.1: Designed `PyUAT` tracking configurations. All configurations define four sets of models that compute likelihoods for specific type of assignment. Values in brackets indicate the mode/mean and scale parameters of the statistical distributions (single value indicates zero mode/mean and only specifies the scale parameter). Tracking configurations that are combined with "`FO` only" show the additional models. Appearance and disappearance models are not shown, but get a value of 0.25 per assignment. The subsampling rate τ is used to adapt the statistical distribution parameters.

Tracking Configuration	Migration Assignment	Cell Division Assignment
Nearest Neighbor<br>(`NN`)	No movement: half-norm($20 \cdot \tau$)<br>No growth: norm($0.05 \cdot \tau$)	No movement: half-norm($20 \cdot \tau$)<br>No growth: norm($0.1 \cdot \tau$)
First-Order<br>(`FO`)	Predict movement: half-norm($20 + 5 \cdot \tau$)<br>Predict size: norm($50 + 10 \cdot \tau$)	Predict movement: half-norm($20 + 2 \cdot 5 \cdot \tau$)<br>Predict size: norm($50 + 2 \cdot 10 \cdot \tau$)
`FO`+orientation<br>(`FO+O`)	Migration angle: half-norm($20 \cdot \tau$)	Division angle: norm(135, $20 \cdot \tau$)
`FO`+distance<br>(`FO+DD`)	-	Division distance: half-norm(3)
`FO`+growth<br>(`FO+G`)	Growth model: norm(1.008^τ, $0.05 \cdot \tau$)	Growth model: norm(1.016^τ, $0.1 \cdot \tau$)
`FO`+orientation+growth+distance<br>(`FO+O+G+DD`)	Predict movement: half-norm($20 + 5 \cdot \tau$)<br>Predict size: norm($50 + 10 \cdot \tau$)<br>Migration angle: half-norm($20 \cdot \tau$)<br>Growth model: norm(1.008^τ, $0.05 \cdot \tau$)	Predict movement: half-norm($20 + 2 \cdot 5 \cdot \tau$)<br>Predict size: norm($50 + 2 \cdot 10 \cdot \tau$)<br>Division angle: norm(135, $20 \cdot \tau$)<br>Division distance: half-norm(3)<br>Growth model: norm(1.016^τ, $0.1 \cdot \tau$)
