# Supplementary material for: PyUAT: An open-source Python framework for uncertainty-aware, efficient, and scalable model-driven cell tracking
Source: PLoS One. 2025 Dec 11;20(12):e0337110. doi: 10.1371/journal.pone.0337110 (PMC12697953; doi:10.1371/journal.pone.0337110)
Supplement: S2 Appendix — (PDF) [file pone.0337110.s002.pdf]

Supplementary Information S2 for

PyUAT: An open-source Python framework for uncertainty-aware, efficient, and scalable model-driven cell tracking

Johannes Seiffarth [1,2] and Katharina Nöh [1,*]

[1] Institute of Bio- and Geosciences, IBG-1: Biotechnology, Forschungszentrum Jülich, 52425 Jülich, Germany

[3] Computational Systems Biotechnology (AVT.CSB), RWTH Aachen University, 52062 Aachen, Germany

*Correspondence: k.noeh@fz-juelich.de

S2.1 Choosing model parameters - Tuning knobs for single-cell behavior modeling

`PyUAT` utilizes tailored models to rate assignments, so that reasonable ones are preferred over nonsensical ones, and combines them in frame-to-frame tracking by utilizing a particle filter approach to iteratively fuse them into full CLTs. The tailored assignment models presented in S1_Appendix allow for statistical modeling of single-cell properties of microbes, for example, their movement or growth behavior. However, all these statistical models and their PDFs come with parameters such as scale or mode. While the mode parameter can be derived from intuition (e.g., no movement) or biological prior knowledge (e.g., expected growth), the scale parameter indicates the spread of the distribution. To investigate the sensitivity of the tracking result derived by `PyUAT` with respect to the choice of the these scale parameters, we selected the first-order tracking configuration and performed a grid search over the scale parameter for the movement and growth models. Figure S2.1 shows the median `TRA` score and execution time evaluated with an imaging interval of 10 minutes. The heatmap shows that the tracking quality is very similar for a wide range of scale parameters. However, the smallest growth and movement scale parameters provide the best tracking result in terms of the `TRA` score. This indicates that the models we designed fit well to the single-cell properties of the studied organism.

Also, the execution time remains very similar over a wide range of scale parameters, except of the region of very low movement scale and high growth scale parameters.

Based on these results, we recommend users to start with large scale parameters and refine them according to the tracking results. This iterative procedure can help to find appropriate parameters tailored to other microbes or cultivation conditions. Manual annotation corrections help to build ground truth data sets to evaluate the tracking performance and find suitable parameters.

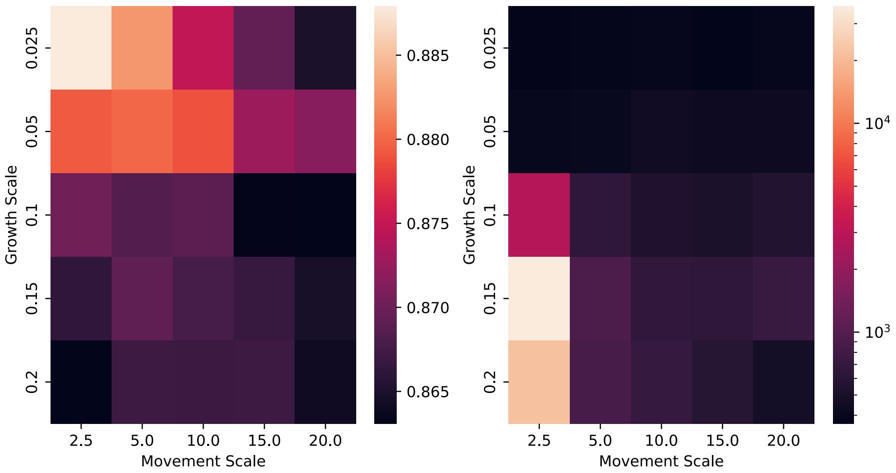

Figure S2.1: Grid search results for growth and movement scale parameters showing TRA score results (left) and tracking execution time in seconds (right).
