# Supplementary material for: PyUAT: An open-source Python framework for uncertainty-aware, efficient, and scalable model-driven cell tracking
Source: PLoS One. 2025 Dec 11;20(12):e0337110. doi: 10.1371/journal.pone.0337110 (PMC12697953; doi:10.1371/journal.pone.0337110)
Supplement: S3 Appendix — (PDF) [file pone.0337110.s003.pdf]

Supplementary Information S3 for

PyUAT: An open-source Python framework for uncertainty-aware, efficient, and scalable model-driven cell tracking

Johannes Seiffarth [1,2] and Katharina Nöh [1,*]

[1] Institute of Bio- and Geosciences, IBG-1: Biotechnology, Forschungszentrum Jülich, 52425 Jülich, Germany

[3] Computational Systems Biotechnology (AVT.CSB), RWTH Aachen University, 52062 Aachen, Germany

[*]Correspondence: k.noeh@fz-juelich.de

S3.1 Tensor Walks: Vectorized walks in cell lineage trees

During the `PyUAT` tracking procedure, quantities need to be extracted from CLTs to score assignment candidates by the assignment models. Therefore, during the execution `PyUAT` often needs to compute walks on the CLT and derive node associated single-cell property development such as position or cell size. To perform the walks in the tree structure efficiently, we developed the `tensor_walks` library that executes vectorized tree walks on a vectorized tree format (see Figure S3.1).

To transfer the CLT into its vectorized form, we first label all nodes with indices, build a tree vector and store the parent of the nodes (or -1 if no parent available). This format allows performing walks within the tree using simple index look-ups in `numpy` and, therefore, makes use of vectorized CPU instructions. Walks are performed in a so called *walk matrix* (see Figure S3.1). We initialize the first row of the walk matrix with the start node indices. With every step, a walk towards the predecessor node is performed using index look-ups in the vectorized CLT. This is continued for a fixed number or until all walks stopped (e.g., due to specified criteria).

We compare the `tensor_walks` execution performance to the widely used and general `networkx` library (`https://github.com/networkx/networkx`). We evaluate the task of estimating the age of a node, that is, the length of its walk to its first predecessor that has a sibling. `tensor_walks` utilizes the vectorized walk computation and `PyUAT` uses standard python loops for computing the age of nodes in a `networkx` graph structure. First benchmarks in sequential trees (i.e., every node index links to the previous index) show a four to five fold acceleration in execution time using the `tensor_walks` library compared to `networkx`, for computing the age of a node in the tree.

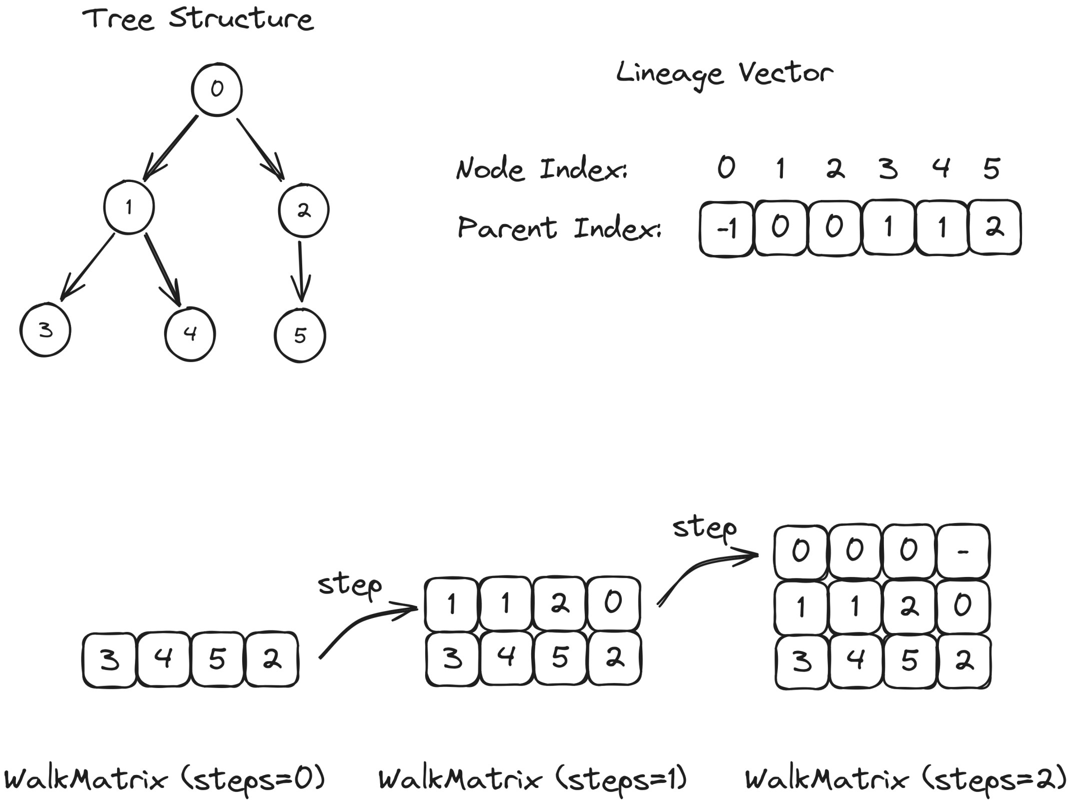

Figure S3.1: Lineage tree example (top left) and its vectorized CLT representation (top right). The nodes are labeled with indices. The lineage vector stores the inverse links of the CLT (left), i.e., for every node the parent index is stored (or -1 if no parent is available). The bottom shows the exemplary walk matrix generation starting at nodes $3, 4, 5, 2$ and performing for two steps. Walks can be reconstructed by traversing the columns of the walk matrix.
