# Supplementary material for: PyUAT: An open-source Python framework for uncertainty-aware, efficient, and scalable model-driven cell tracking
Source: PLoS One. 2025 Dec 11;20(12):e0337110. doi: 10.1371/journal.pone.0337110 (PMC12697953; doi:10.1371/journal.pone.0337110)
Supplement: S4 Appendix — (PDF) [file pone.0337110.s004.pdf]

Supplementary Information S4 for

PyUAT: An open-source Python framework for uncertainty-aware, efficient, and scalable model-driven cell tracking

Johannes Seiffarth [1,2] and Katharina Nöh [1,*]

[1] Institute of Bio- and Geosciences, IBG-1: Biotechnology, Forschungszentrum Jülich, 52425 Jülich, Germany

[3] Computational Systems Biotechnology (AVT.CSB), RWTH Aachen University, 52062 Aachen, Germany

[*]Correspondence: k.noeh@fz-juelich.de

S4.1 Computing optimal frame-to-frame lineages using integer linear programming

`PyUAT` utilizes a particle filter approach to iteratively combine frame-to-frame lineages into a CLT that covers the full time-lapse. To compute the frame-to-frame lineages `PyUAT` uses an integer linear program (ILP) formulation. In the following we describe the construction of the frame-to-frame ILP.

Let $A_{t:t+1} = \{a_1, a_2, \ldots\}$ be the set of assignment candidates that are considered for connecting cell detections $D_t = \{d_1^t, \ldots, d_{N_t}^t\}$ at frame t to cell detections $D_{t+1} = \{d_1^{t+1}, \ldots, d_{N_{t+1}}^{t+1}\}$ at the next frame $t+1$. The likelihood of an assignment $a \in A_{t:t+1}$ is computed by

$$p(a) = \prod_{m \in MS(a)} p_m(a) \quad \in [0,1] \tag{S4.1}$$

where $MS(a) = \{m_1(\cdot), m_2(\cdot), \ldots\}$ is the set of assignment models with probability functions $p_m(\cdot)$ that are used for rating the type of assignment (see S1_Appendix).

To represent the selection of all assignments, we define the boolean vector $\vec{b} \in \{0,1\}^{|A_{t:t+1}|}$ that contains a boolean entry for every assignment candidate between frames t and $t+1$, where an entry of 1 denotes that the assignment is part of the selection $\vec{b}$, whereas a value of 0 indicates that the assignment is not part of the selection $\vec{b}$. The selection $\vec{b}$ represents a valid frame-to-frame tracking, if and only if every detection at frame t and $t+1$ is part of exactly one selected assignment. This is expressed in terms of linear constraints

$$\forall d^t \in D_t \quad : \quad \sum_{i=0}^{|A^{t:t+1}|} \mathbb{1}(d^t, a_i) = 1 \tag{S4.2}$$

$$\forall d^{t+1} \in D_{t+1} \quad : \quad \sum_{i=0}^{|A^{t:t+1}|} \mathbb{1}(d^{t+1}, a_i) = 1 \tag{S4.3}$$

where $\mathbb{1}(\cdot,\cdot) \in \{0,1\}$ indicates that detection d is part of assignment a or not:

$$\mathbb{1}(d,a) = \begin{cases} 1, & \text{if detection } d \text{ is part of assignment } a \\ 0, & \text{otherwise} \end{cases} \tag{S4.4}$$

The most likely frame-to-frame tracking $\vec{b}_{opt}$ is computed by maximizing the joint probability of the selected assignments, while satisfying the conditions in Eqs. (S4.2)-(S4.3):

$$\vec{b}_{opt} = \underset{\vec{b} \in \{0,1\}^{|A_{t:t+1}|}}{\operatorname{argmax}} \prod_{i=0}^{|A_{t:t+1}|} p(a_i)^{b_i}. \tag{S4.5}$$

By applying the logarithm to the objective function, we reformulate the optimization problem in terms of a linear objective function

$$\vec{b}_{opt} = \underset{\vec{b} \in \{0,1\}^{|A_{t:t+1}|}}{\operatorname{argmax}} \prod_{i=0}^{|A_{t:t+1}|} p(a_i)^{b_i} = \underset{\vec{b} \in \{0,1\}^{|A_{t:t+1}|}}{\operatorname{argmax}} \sum_{i=0}^{|A_{t:t+1}|} b_i \cdot \log p(a_i). \tag{S4.6}$$

We solve this ILP for the optimal frame-to-frame tracking $\vec{b}_{opt}$ using `Gurobi` or `CBC`.
