# Supplementary material for: PyUAT: An open-source Python framework for uncertainty-aware, efficient, and scalable model-driven cell tracking
Source: PLoS One. 2025 Dec 11;20(12):e0337110. doi: 10.1371/journal.pone.0337110 (PMC12697953; doi:10.1371/journal.pone.0337110)
Supplement: S5 Appendix — (PDF) [file pone.0337110.s005.pdf]

Supplementary Information S5 for

PyUAT: An open-source Python framework for uncertainty-aware, efficient, and scalable model-driven cell tracking

Johannes Seiffarth [1,2] and Katharina Nöh [1,*]

[1] Institute of Bio- and Geosciences, IBG-1: Biotechnology, Forschungszentrum Jülich, 52425 Jülich, Germany

[3] Computational Systems Biotechnology (AVT.CSB), RWTH Aachen University, 52062 Aachen, Germany

[*] Correspondence: k.noeh@fz-juelich.de

S5.1 Sub-sampled dataset sizes

The tracking method is evaluated for different sub-sampling factors. Applying these sub-sampling factors to the Tracking-one-in-a-million (TOIAM) dataset [1] mimics an increase in image frame rates. It also changes the number of images, cell detections, and number of cell tracks. Table S5.1 shows the size statistics for the sub-sampled datasets.

Sub-sampling factor	Number of images	Number of cell detections	Number of cell tracks
1	4,000	1,415,272	29,863
2	2,000	705,699	29,530
3	1,335	471,792	29,416
4	1,000	350,841	28,965
5	800	280,041	28,681
6	670	237,803	29,102
7	575	204,333	28,997
8	500	173,664	27,993
9	445	154,741	27,929
10	400	138,166	27,493
13	310	108,893	27,749
16	250	84,909	26,131
19	215	77,707	28,074
22	185	65,526	26,998
25	160	53,032	24,262
30	135	46,058	24,645
35	115	37,813	22,928
40	100	31,762	21,401

Table S5.1: Size statistics of the sub-sampled TOIAM dataset. The sub-sampling factor corresponds to the minutes between frames. Each row shows the cumulative statistics for all five time-lapses.

References

1. Seiffarth J, Blöbaum L, Paul RD, Friederich N, Sitcheu AJY, Mikut R, et al. Tracking One-in-a-Million: Large-Scale Benchmark for Microbial Single-Cell Tracking with Experiment-Aware Robustness Metrics. In: Computer Vision – ECCV 2024 Workshops; 2025. p. 318–334. Available from: https://doi.org/10.1007/978-3-031-91721-9_20.
